# Independent Evolution of Six Families of Halogenating Enzymes

**DOI:** 10.1371/journal.pone.0154619

**Published:** 2016-05-06

**Authors:** Gangming Xu, Bin-Gui Wang

**Affiliations:** Key Laboratory of Experimental Marine Biology, Institute of Oceanology, Chinese Academy of Sciences, Nanhai Road 7, Qingdao, 266071, People’s Republic of China; Weizmann Institute of Science, ISRAEL

## Abstract

Halogenated natural products are widespread in the environment, and the halogen atoms are typically vital to their bioactivities. Thus far, six families of halogenating enzymes have been identified: cofactor-free haloperoxidases (HPO), vanadium-dependent haloperoxidases (V-HPO), heme iron-dependent haloperoxidases (HI-HPO), non-heme iron-dependent halogenases (NI-HG), flavin-dependent halogenases (F-HG), and *S*-adenosyl-L-methionine (SAM)-dependent halogenases (S-HG). However, these halogenating enzymes with similar biological functions but distinct structures might have evolved independently. Phylogenetic and structural analyses suggest that the HPO, V-HPO, HI-HPO, NI-HG, F-HG, and S-HG enzyme families may have evolutionary relationships to the α/β hydrolases, acid phosphatases, peroxidases, chemotaxis phosphatases, oxidoreductases, and SAM hydroxide adenosyltransferases, respectively. These halogenating enzymes have established sequence homology, structural conservation, and mechanistic features within each family. Understanding the distinct evolutionary history of these halogenating enzymes will provide further insights into the study of their catalytic mechanisms and halogenation specificity.

## Introduction

Halogenated compounds are widespread in nature, particularly in marine environment, and they have diverse structures and versatile bioactivities [1–3]. The halogen substituent is typically critical to the bioactivities of these compounds. There is great interest in the biosynthesis and mechanistic studies of halogenated compounds, including the antibiotics vancomycin and chloramphenicol, the antioxidants bromophenols, and the antitumor agents rebeccamycin and salinosporamides [4–6]. Previous studies have revealed that these halogenated natural products were biosynthesized by halogenating enzymes [5, 7]. A few examples of halogenated natural products catalyzed by different halogenating enzymes are shown in S1 Fig. According to their cofactor dependence, six families of halogenating enzymes have been reported so far **(**S1 Table**)**, including cofactor-free haloperoxidases (HPO), vanadium-dependent haloperoxidases (V-HPO), heme iron-dependent haloperoxidases (HI-HPO), non-heme iron-dependent halogenases (NI-HG), flavin-dependent halogenases (F-HG), and *S*-adenosyl-L-methionine (SAM)-dependent halogenases (S-HG) [7–9].

In the cofactor-free HPO enzyme family, only five crystal structures have been determined, all from bacterial species [9]. These structures include the chloroperoxidases CPO-A1, CPO-A2, and CPO-T from *Streptomyces aureofaciens*, CPO-L from *S*. *lividans*, and CPO-F from *Pseudomonas fluorescens*. Although CPO-A2 has a catalytic triad, other halogenation mechanism was proposed. The cofactor-free HPO enzymes have an active site pocket, besides the specific halide-binding sites. These enzymes require organic acids, such as benzoate or propionate, as cosubstrate for their bioactivity [9, 10].

The V-HPO family has the most well-studied halogenating enzymes from algae, fungi, and bacteria [11, 12]. The V-HPO enzymes use vanadium (V) as a cofactor and catalyze the oxidation of halides (Cl, Br and I) using hydrogen peroxide (H_2_O_2_). Several crystal structures of V-HPO enzymes have been reported, including V-bromoperoxidases (V-BPO), V-chloroperoxidases (V-CPO), and V-iodoperoxidases (V-IPO) [12]. The structure-function relationships of the V-HPO enzymes are relatively well understood and reviewed very recently [12, 13].

The HI-HPO family consists of heme-thiolate enzymes that catalyze halogenation reactions in the presence of H_2_O_2_ [14]. Several crystal structures of HI-HPO enzymes with their respective substrates from fungi and mammals have been determined [14–16], The fungal chloroperoxidase (CPO) from *Caldariomyces fumago* is involved in caldarioymcin biosynthesis [14]. The HI-HPO family also includes mammalian enzymes, such as human myeloperoxidase (MPO) and animal lactoperoxidase (LPO) [15, 17]. The conserved halide-binding sites and bound substrates of some HI-HPO enzymes were also determined [14–16].

The NI-HG family is a class of highly homologous enzymes that halogenate amino acid methyl groups, using non-heme iron (Fe^2+^), O_2_ and α-ketoglutarate (α-KG) as cofactors [18]. Four enzymes of the NI-HG family have been well characterized [18–21]. CmaB (*P*. *syringae*) is involved in chlorinating the γ-position of L-*allo*-isoleucine during coronamic acid biosynthesis [18]. SyrB2 (*P*. *syringae*) is involved in the chlorination of threonine during the biosynthesis of syringomycin E [19]. CytC3 (*Streptomyces* sp.) catalyzes a double chlorination reaction during the *γ*,*γ*-dichloroaminobutyrate biosynthesis [20]. CurA-Hal (*Lyngbya majuscula*) catalyzes the cyclopropane ring formation of the curacin A [21]. After the crystal structures of SyrB2, CytC3 and CurA-Hal were determined, their halogenating mechanisms were also extensively studied [22–24].

All of the F-HG family enzymes contain a conserved flavin (FADH_2_) binding site, and six of them have crystal structures reported [8]. PyrH (*S*. *rugosporu*) is a regioselective tryptophan 5-halogenase during pyrroindomycin biosynthesis [25]. PrnA (*P*. *fluorescens*) is a tryptophan 7-halogenase with regioselective chlorination during pyrrolnitrin biosynthesis [26]. RebH (*Lechevalieria aerocolonigenes*) is also a tryptophan 7-halogenase, showing regioselective arene halogenation during rebeccamycin synthesis [27]. CmlS (*S*. *venezuelae*) has a covalent flavin-aspartate bond and is involved in chloramphenicol biosynthesis [28]. CndH (*Chondromyces crocatus*) is a myxobacterial chondrochloren halogenase of a new variant group [29]. PltA (*P*. *fluorescens*) is a FADH_2_-dependent halogenase that catalyzes dichlorination during pyoluteorin biosynthesis [30].

The S-HG family contains SAM-dependent halogenating enzymes that use a nucleophilic substitution mechanism [31]. The 5'-fluoro-5'-deoxyadenosine synthase (FDAS) from *Streptomyces cattleya* is the first native fluorinating enzyme discovered that catalyzes the formation of a C-F bond using nucleophilic substitution [32, 33]. SalL (*Salinispora tropica*) is a marine bacterial SAM-dependent chlorinase that is involved in the biosynthesis of the anticancer agent salinosporamide A [34]. The fluorinase FDAS also has chlorinase activity; whereas the chlorinase SalL also utilizes bromide and iodide as substrates [34, 35].

These six families of halogenating enzymes have similar biological functions, but the various enzyme structures suggest that they might have divergent evolutionary processes. It was suggested that the V-HPO family have evolutionary relationship to the acid phosphatases [11, 12]. Based on the crystal structures and catalytic mechanisms, phylogenetic and structural analyses suggest that these halogenating enzymes may be evolutionarily related to other enzymes. The elucidation of their divergent evolutionary history will be helpful for the future investigation of their halogenating mechanisms and substrate selectivity.

## Materials and Methods

All the halogenating enzyme homologues with biological relevance were obtained using the position-specific iterated (PSI)-BLAST in the GenBank [36]. The conserved domain database (CDD) and the Pfam protein families database for putative conserved domains were also used [37, 38]. Distant homologues with similar structural macromolecular complexes were also tracked by the vector alignment search tool (VAST+) in the molecular modeling database (MMDB), and by the FUGUE 2.0 program using sequence-structure comparisons in the protein data bank (PDB) [39, 40]. The multiple sequence alignment (MSA) was aligned using the MUSCLE program in the MEGA 6.0 suite [41]. The structure-based MSA analyses were performed and visualized using the ESPript 3.0 program [42]. The predictions of protein quaternary structure were performed using the SWISS-MODEL homology-modeling server [43]. The 3D molecular structures of enzymes were visualized and prepared using the PyMOL molecular graphics system.

The reconstruction of phylogenetic trees was performed using neighbor-joining (NJ), minimum-evolution (ME), and maximum likelihood (ML) methods [44]. The phylogeny was tested by the bootstrap and interior-branch methods with 1000 replications. The substitution models were analyzed using the p-distance model for NJ trees and the Poisson model with gamma distributed correction for NJ, ME and ML methods in the MEGA 6.0 suite [45]. The gaps/missing data treatment is using partial deletion [41]. The ML trees were firstly to find best models and then reconstructed using the MEGA 6.0 software [45]. Similar topologies were obtained from different methods, and only the most robust phylogenetic trees from the NJ methods are shown.

## Results and Discussion

### The HPO family relationships to the α/β hydrolases

**Fig 1:**
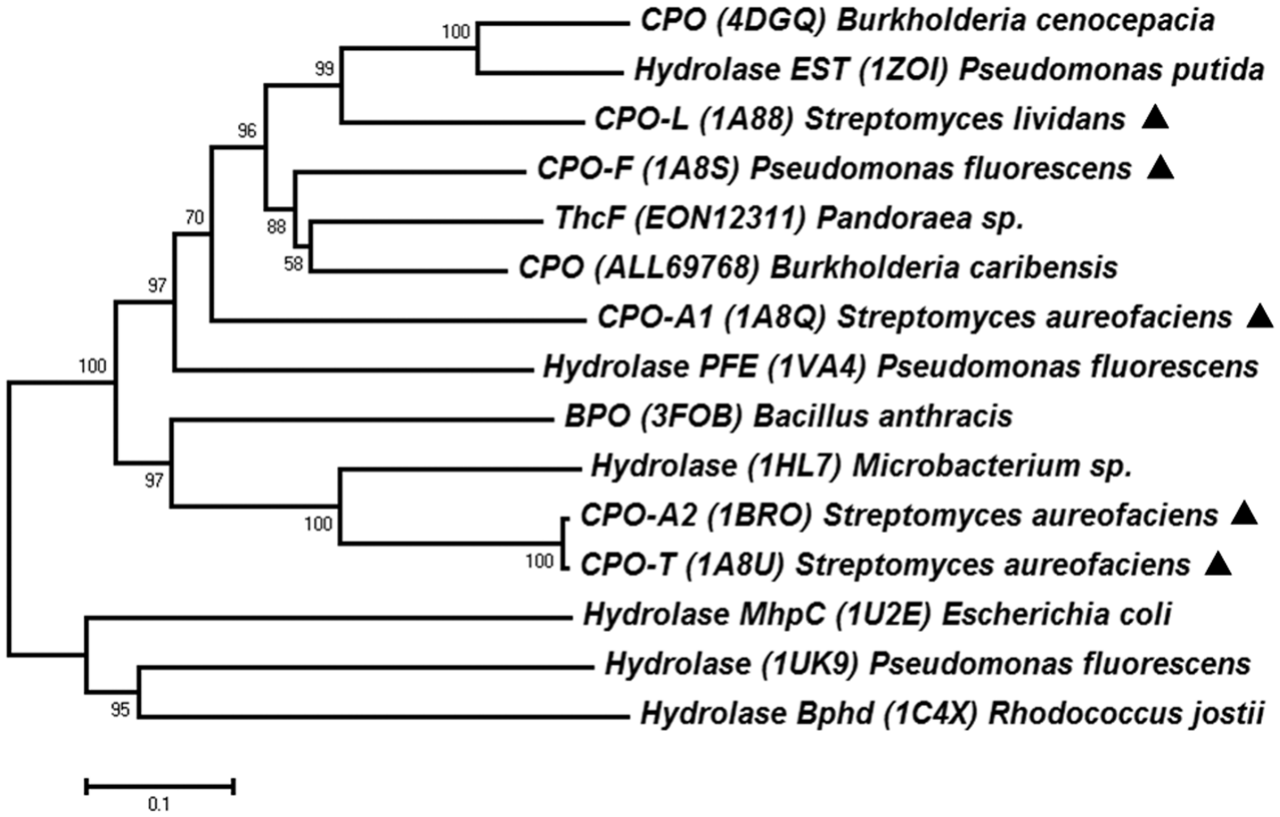
Evolutionary relationships between the cofactor-free HPO and the α/β hydrolases. The phylogenetic tree was reconstructed using the Neighbor-Joining method. The representative HPO enzymes are marked (▲).

The crystal structures of five cofactor-free HPO family enzymes (CPO-A1, CPO-A2, CPO-T, CPO-L and CPO-F) from bacteria have been determined [9]. These HPO enzymes share more than 39% sequence identity. The HPO structures showed great similarity with the general topology of the α/β hydrolase fold [9, 10]. In order to further understand their evolutionary relationships, these HPO enzyme homologues (particularly distant homologues) were obtained from the GenBank and the PDB databases. After MSA analysis, phylogenetic trees were reconstructed using various methods. As shown in Fig 1, these HPO family enzymes clustered separately, some of them with homologues of the α/β hydrolase family. For example, CPO-A2 and CPO-T (*S*. *aureofaciens*) closely clustered with the hydrolase 1HL7 (*Microbacterium* sp.); and the CPO (*Burkholderia cenocepacia*) appears to be closely related to the hydrolase EST (*P*. *putida*) [46]. Some remote homologues of the α/β hydrolase family clustered as a separate clade, such as the hydrolases MhpC (*Escherichia coli*) and Bphd (*Rhodococcus jostii*) [47].

Structure-based MSA analysis (S2 Fig) showed that the structures of the HPO enzymes also have the featured α/β hydrolase fold with a catalytic triad (S98-D228-H257) in the active sites for halogenation [10]. Comparing the general scheme of the HPO enzymes and the α/β hydrolases, we found that the central β-sheet and six covering helices are mostly conserved. Additional conserved residues included the GYR and DRRG motifs, and also several other residues (HG, D-G-G-S and G-S-G-G) for structure arrangement and stabilization [9, 48]. The comparison between crystal structures of the HPO enzymes and the α/β hydrolases suggest that they all have similar overall topology, which is featured with the α/β hydrolase fold (S3 Fig). The α/β hydrolase family also contains some esterases and lipases, which are structurally related but have diverse substrate specificity [49]. These enzymes have conserved arrangement of catalytic triad residues and structural features, and may share a common ancestor [50, 51]. The cofactor-free HPO enzymes may need organic acids as cosubstrates, such as CPO-T with benzoate and CPO-F with propionate [9]. The reaction mechanism may involve a conserved substrate-binding pocket for hydrophobic compounds halogenation at specific carbon center. These HPO enzymes may catalyze chlorination and bromination reactions, although no halogenated natural products have been proved yet [9]. Based on the phylogenetic clusters and structural similarities, the cofactor-free HPO family might have evolutionary relationships with the α/β hydrolases, with similar substrates and bioactivities.

### The V-HPO family relationships to the acid phosphatases

Crystal structures of the V-BPO, V-CPO, and V-IPO enzymes (sequence identity 17~33%) have been determined from brown and red algae, fungi, and bacteria [13, 52–54]. The V-HPO enzymes can catalyze chlorination, bromination, and iodination reactions, with different efficiency [12]. Previous studies suggest that the V-HPO family may have relationships to the acid phosphatases [11, 12, 55]. Phylogenetic analysis of the V-HPO homologues showed that the V-HPO enzymes from different origins clustered closely together (S4 Fig). It suggests that these enzymes might be derived from a common ancestor. During the course of divergent evolution, the bacterial V-HPO enzymes may become clustered independently and have similarities to the type 2 phosphatidic acid phosphatase (PAP2) family [55]. The PAP2 superfamily includes the bacterial non-specific acid phosphatases and a variety of HPO enzymes, which may share a similar evolutionary history [12, 56].

Structure-based MSA analysis showed that the V-HPO enzymes contain many α-helices, and the C-terminal has several conserved sites (S5 Fig). Despite a relatively low level of sequence identity, several short motifs in the C-terminal were still highly conserved between the V-HPO enzymes and acid phosphatases, including the cofactor vanadate coordination sites RP, Y-SGH, and R-G-H-D [12, 13]. The overall shape of the V-HPO family monomer enzyme looks like a cylinder, with a variable N-terminal helix-bundle and a conserved C-terminal helix-bundle (S6 Fig). The most conserved sites for VO_4_ coordination were located at the end of the C-terminal helix-bundle [12]. The non-specific acid phosphatase, such as EB-NSAP from *Escherichia blattae*, has only one helix-bundle motif, which contains 5 helixes [55]. There is also great similarity in the structural fold between the V-HPO and the acid phosphatases [11, 12]. Phylogenetic analyses and structural similarities suggest that the V-HPO enzymes and the acid phosphatases are evolutionarily related, which may share a common ancestor. Gene duplication and fusion might have played a vital role during the divergent evolution of the V-HPO family [12, 13]. The significance of these events to function is that they may have similar bioactivity, due to the high similarity in structure and cofactor-binding manner [12]. For example, the V-CPO (*C*. *inaequalis*) was reported to exhibit phosphatase activity [57]. These all suggest that the V-HPO family may have evolutionary and functional relationships to the acid phosphatases [11, 12].

### The HI-HPO family relationships to the peroxidases

**Fig 2:**
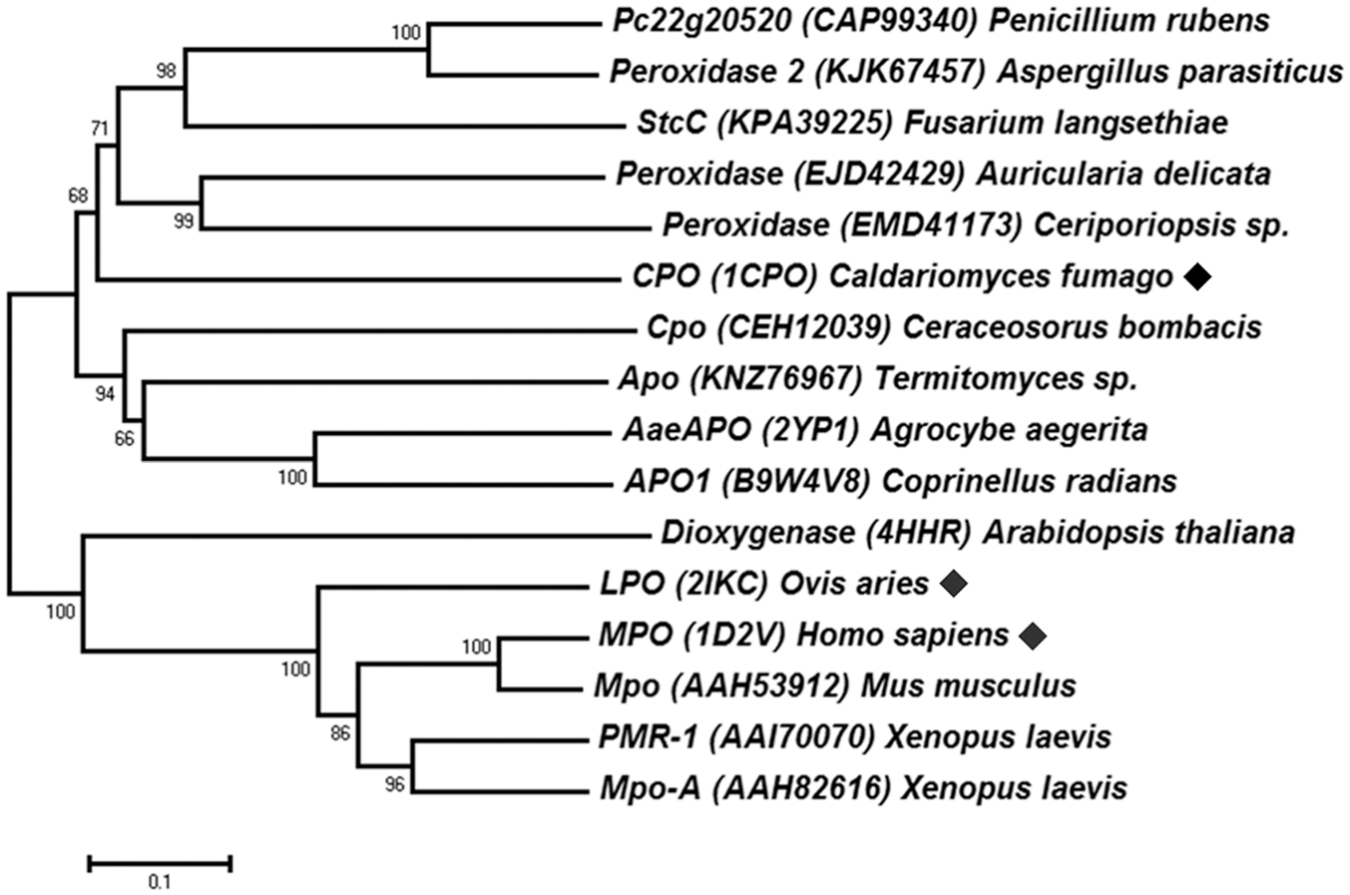
Evolutionary relationships between the HI-HPO and the peroxidases. The phylogenetic tree was reconstructed using the Neighbor-Joining method. The representative HI-HPO enzymes are marked (◆).

Only one enzyme crystal structure of the HI-HPO family has been determined from fungi, the fungal CPO from *C*. *fumago* [14, 58]. Phylogenetic trees were reconstructed after MSA analysis of CPO homologues, and the NJ tree is shown in Fig 2. The CPO (*C*. *fumago*) was closely clustered with other peroxidase family members. The peroxidase family 2 also includes cytochrome P450-like oxygenases; whereas the CPO was proved to be a functional hybrid of peroxidase-P450 [58]. The relatively close homologue (sequence identity 24%) is the fungal enzyme AaeAPO from *Agrocybe aegerita*. AaeAPO was the first reported aromatic peroxygenases, which also represents an evolutionary link between heme peroxidases and P450 [16]. Other distant homologues clustered separately, including the mammalian heme peroxidases MPO and LPO [15, 17]. The HI-HPO enzymes could oxidize the halides with a lower electronegativity. For example, the CPO may also accepts bromide and iodide [7].

Structure-based MSA analysis of the HI-HPO and peroxidases (S7 Fig) suggest that the highly conserved regions include: the proximal heme-binding motif (D-R-PCP-N-LA-H) at the N-terminal, the E-D-S motif for heme propionates in the middle, and the acid-base catalyst E residue in the distal heme pocket [59, 60]. The specific residues in the substrate binding pocket of these enzymes might be variable to ensure their catalytic specificity [14, 16]. Comparison of the overall structures between the HI-HPO and the peroxidases also showed some structural similarities (S8 Fig). These enzymes are rich in α-helixes, particularly in the highly conserved core heme-binding site and the halide-binding pocket [16]. Moreover, their catalytic properties are also somewhat similar. For example, the typical CPO (*C*. *fumago*) has catalase and P450-like monooxygenase activity; whereas the predominant peroxygenase AaeAPO (*A*. *aegerita*) also displayed weak bromoperoxidase activity [14, 16, 61]. These indicate that there might be evolutionary and functional relationships between the HI-HPO enzymes and the peroxidases.

### The NI-HG family relationships to the chemotaxis phosphatases

**Fig 3:**
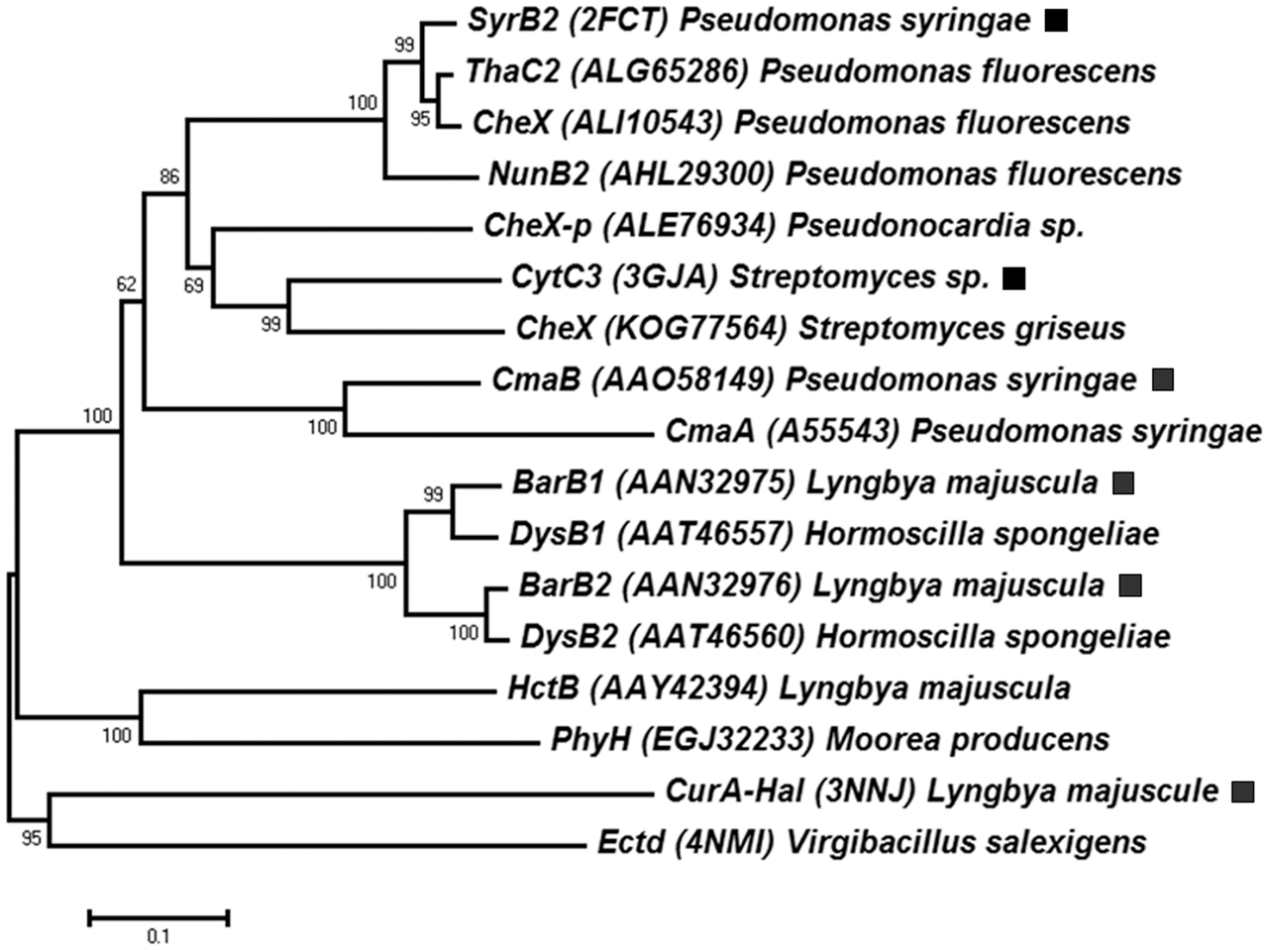
Evolutionary relationships between the NI-HG and the chemotaxis phosphatases. The phylogenetic tree was reconstructed using the Neighbor-Joining method. The representative NI-HG enzymes are marked (■).

Four chlorinases of the NI-HG family have been characterized, including CmaB (*P*. *syringae*), SyrB2 (*P*. *syringae*), CytC3 (*Streptomyces* sp.), and CurA-Hal (*Lyngbya majuscula*), which could also catalyze bromination [18–20, 24]. These NI-HG enzymes share about 20~57% sequence identity. Phylogenetic trees were reconstructed using these protein homologues, and the NJ tree is shown in Fig 3. The NI-HG homologues also include BarB1 and BarB2, which is involved in a trichlorination during the biosynthesis of barbamide in the cyanobacterium *Lyngbya majuscula* [62]. Surprisingly, the BlastP search of the SyrB2 and CytC3 homologues also revealed many chemotaxis proteins CheX in the GenBank database. The CheX protein is one of the phosphatases in the chemotaxis signal transduction system of bacteria [63, 64]. Some of the CheX coding genes (such as ALE76934) are located on plasmids, which suggest that horizontal gene transfer might play a role during the evolutionary process.

Structure-based MSA analysis (S9 Fig) showed that the NI-HG family has conserved motifs (HLD-H and HQA-H) for iron coordination, and also contains conserved active site residues (H, F, R and S) in a hydrophobic pocket for chloride binding [19]. The overall structures of the NI-HG enzymes are slightly different from the chemotaxis protein CheX; however, the conserved fold might be similar in these distant homologues (S10 Fig). Within the core structure of the NI-HG enzymes is a *β*-sandwich ‘jelly-roll’ motif (cupin fold) composed of eight anti-parallel strands [19, 20]. The chemotaxis proteins of the CheX family were reported to have a conserved (E-xx-N) motif; whereas only crystal structures of CheX in *Thermotoga maritima* and *Borrelia burgdorferi* have been determined [63, 64]. No other close homologue between the two families has been reported, and therefore more experimental data regarding the catalytic mechanisms and evolutionary relationships are needed.

### The F-HG family relationships to the oxidoreductases

**Fig 4:**
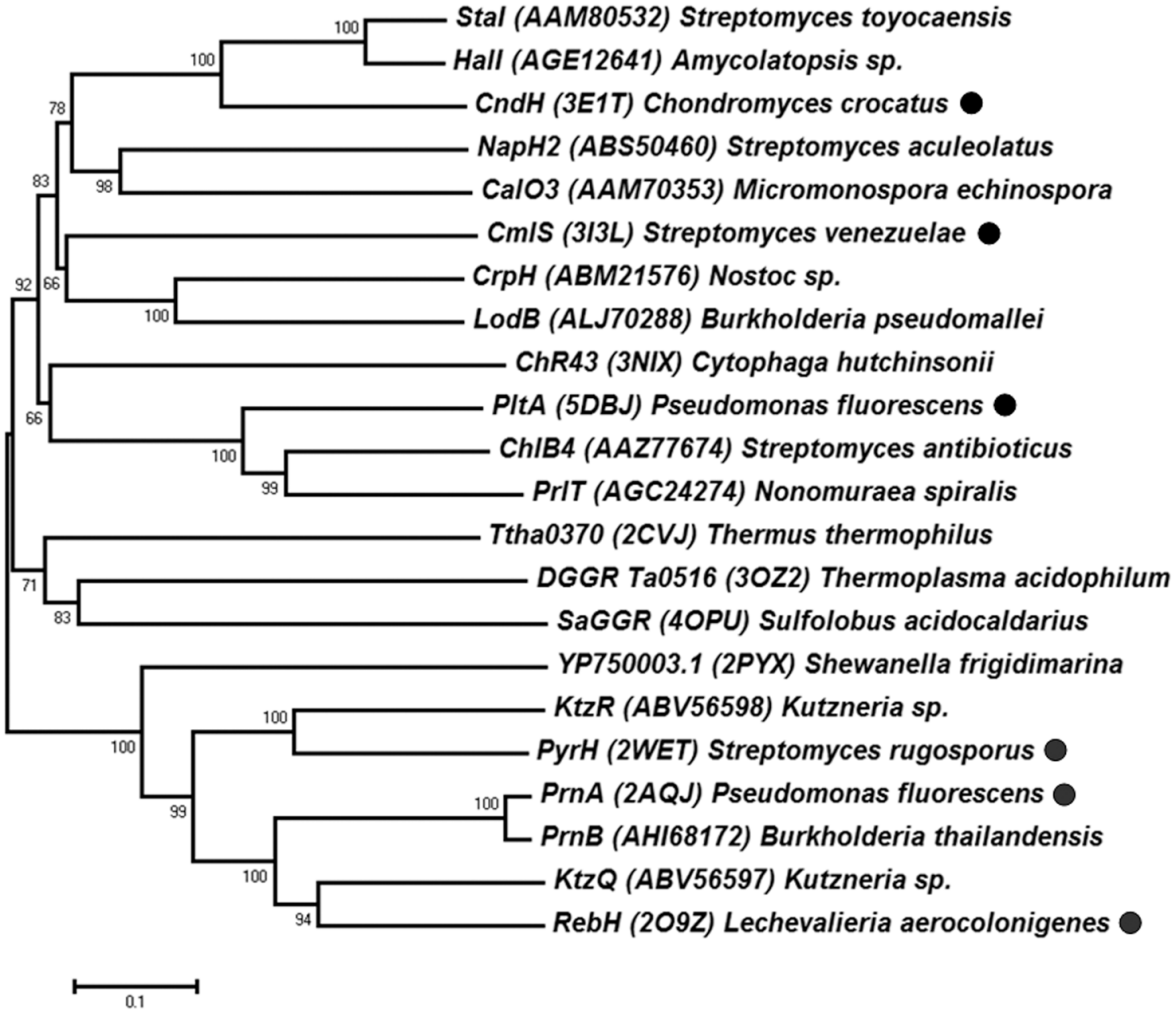
Evolutionary relationships between the F-HG and the oxidoreductases. The phylogenetic tree was reconstructed using the Neighbor-Joining method. The representative F-HG enzymes are marked (●).

The F-HG family enzymes all contain a conserved flavin-binding fold [8]. Thus far, six halogenase crystal structures have been determined, including PyrH (*S*. *rugosporu*), PrnA (*P*. *fluorescens*), RebH (*L*. *aerocolonigenes*), CmlS (*S*. *venezuelae*), CndH (*C*. *crocatus*), and PltA (*P*. *fluorescens*) [8, 25–29]. These F-HG enzymes share about 20~56% sequence identity. Phylogenetic trees were reconstructed using the close and distant homologues of the F-HG enzymes. As shown in Fig 4, the F-HG family enzymes form two main subgroups. The PyrH, PrnA, and RebH were categorized as tryptophan halogenases; the CmlS, CndH, and PltA were categorized as non-tryptophan halogenases. The F-HG enzymes were classified based on their halogenation substrates: variant A enzymes utilize free small molecules like tryptophan; variant B enzymes catalyze substrates that bind as a thioester [8, 28]. The tryptophan halogenases clustered closely together on a separate branch; whereas the non-tryptophan halogenases clustered on different sub-branches. These differences in clustering may be due to the substrate specificity of these enzymes. There might be no clear boundary between the two variants [8]. All of the F-HG enzymes reported are chlorinases, which can also function as brominases [7, 65, 66]. Moreover, distant homologues of the F-HG enzymes may also include some members of the glutathione reductase (GR) superfamily [8, 67].

Structure-based MSA analysis (S11 Fig) showed that the F-HG family enzymes have a conserved Rossmannoid-fold FAD-binding domain, consisting of a β-sheet flanked by helixes. All the F-HG enzymes contain an N-terminal G-box (GxGxxG) motif, which is also conserved in the GR superfamily [8, 67]. There is also a conserved WxWxIP motif in the C-terminal, and several G residues are conserved for hydrogen bonding with FAD. The C-terminal domain is variable, and contains the active site residues responsible for specific substrate binding [8, 28]. Interestingly, the VAST+ search for structurally similar homologues of the F-HG family enzymes identified several oxidoreductases (sequence identity > 20%), including ChR43 (*Cytophaga hutchinsonii*), SaGGR (*Sulfolobus acidocaldarius*), Ta0516 (*Thermoplasma acidophilum*), and Ttha0370 (*Thermus thermophilus*) [8]. These enzymes have very similar overall structures, although the C-terminal domains are different (S12 Fig). Phylogenetic and structural analyses suggest that there might be evolutionary relationships between the F-HG family and the oxidoreductases.

### The S-HG family relationships to the SAM hydroxide adenosyltransferases

**Fig 5:**
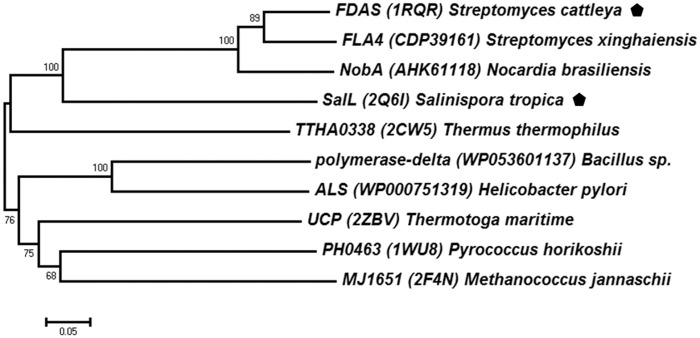
Evolutionary relationships between the S-HG and the SAM hydroxide adenosyltransferases. The phylogenetic tree was reconstructed using the Neighbor-Joining method. The representative S-HG enzymes are marked (pentagon).

Only two crystal structures of the S-HG enzymes have been reported, the fluorinase FDAS (*Streptomyces cattleya*) and the chlorinase SalL (*Salinispora tropica*), which share 38% sequence identity [32, 34]. Homologues of these two S-HG enzymes were searched in the GenBank database, and only a few amino acid sequences were identified (Fig 5). One close homologue is NobA (*Nocardia brasiliensis*), which has 79% and 36% sequence identity to FDAS and SalL, respectively. Interestingly, several distant homologues of the SAM hydroxide adenosyltransferases (PF01887, previously known as duf-62 family) were also found, such as PH0463 (*Pyrococcus horikoshii*) and MJ1651 (*Methanococcus jannaschii*) [68, 69]. The SAM hydroxide adenosyltransferases catalyze a hydrolytic cleavage of SAM to generate adenosine and L-methionine. Similar S_N_2 nucleophilic substitution was employed, with the replacement of the halide ion by water or hydroxide [31].

Structure-based MSA analysis (S13 Fig) showed that two motifs (PxNGL and IDxxFGN), and several active site residues (such as D16 and N215) are conserved. The conserved residues in the binding pocket might form hydrogen bonds with substrate [31]. However, the key residues for the halide co-ordination are different. The S-HG family enzymes use T80-S158 in FDAS and Y70-G131 in SalL [32, 34]. The SAM hydroxide adenosyltransferases utilize a conserved amino acid triad (D68-R75-H127), which may activate water to hydroxide ions [68]. The variable organization of the active site may ensure their substrate specificity and reaction in different manners. These enzymes also have very similar tertiary structures, which are asymmetric homotrimer [31]. Each monomer unit has two domains: the N-terminal α/β/α sandwich domain and the C-terminal anti-parallel β-sheet domain (S14 Fig). However, these enzymes may have different biological functions. The fluorinase FADS could also act as a chlorinase [35]. The chlorinase SalL also accepts Br^-^ and I^-^, but not F^-^ [34]. The SAM hydroxide adenosyltransferases utilize water/hydroxide, but no halogenation activity [68]. Therefore, phylogenetic and structural analyses suggest the S-HG enzymes might be evolutionarily related to the SAM hydroxide adenosyltransferases.

In summary, phylogenetic and structural analyses suggest that there might be evolutionary and functional relationships between the HPO and the α/β hydrolases, the V-HPO and the acid phosphatases, the HI-HPO and the peroxidases, the NI-HG and the chemotaxis phosphatases, the F-HG and the oxidoreductases, and the S-HG and the SAM hydroxide adenosyltransferases, respectively. These enzymes have established conserved sequence, structural, and mechanistic features within each family. As summarized in the S1 Fig, by comparison of the halogenated natural products and the halogenating enzymes, and also considering the phylogenetic clusters of each family, it is possible to map different halogen specificities to different families. Understanding the distinct evolutionary process might be helpful for the study of their biological function and halogenation specificity.

## Supporting Information

S1 Fig Examples of halogenated natural products with their proposed specific halogenating enzymes. (PDF)

S2 Fig Multiple sequence alignment of the cofactor-free HPO and the α/β hydrolases. (PDF)

S3 Fig Structure comparison of the cofactor-free HPO and the α/β hydrolases. (PDF)

S4 Fig Evolutionary relationships between the V-HPO and the acid phosphatases. (PDF)

S5 Fig Multiple sequence alignment of the V-HPO and the acid phosphatases. (PDF)

S6 Fig Structure comparison of the V-HPO and the acid phosphatase. (PDF)

S7 Fig Multiple sequence alignment of the HI-HPO and the peroxidases. (PDF)

S8 Fig Structure comparison of the HI-HPO and the peroxidases. (PDF)

S9 Fig Multiple sequence alignment of the NI-HG and the chemotaxis phosphatases. (PDF)

S10 Fig Structure comparison of the NI-HG and the chemotaxis phosphatase. (PDF)

S11 Fig Multiple sequence alignment of the F-HG and the oxidoreductases. (PDF)

S12 Fig Structure comparison of the F-HG and the oxidoreductases. (PDF)

S13 Fig Multiple sequence alignment of the S-HG and the SAM hydroxide adenosyltransferases. (PDF)

S14 Fig Structure comparison of the S-HG and the SAM hydroxide adenosyltransferases. (PDF)

S1 Table Six families of halogenating enzymes. (PDF)
