# Supplementary material for: Independent Evolution of Six Families of Halogenating Enzymes
Source: PLoS One. 2016 May 6;11(5):e0154619. doi: 10.1371/journal.pone.0154619 (PMC4859513; doi:10.1371/journal.pone.0154619)
Supplement: S1 Fig — (PDF) [file pone.0154619.s001.pdf]

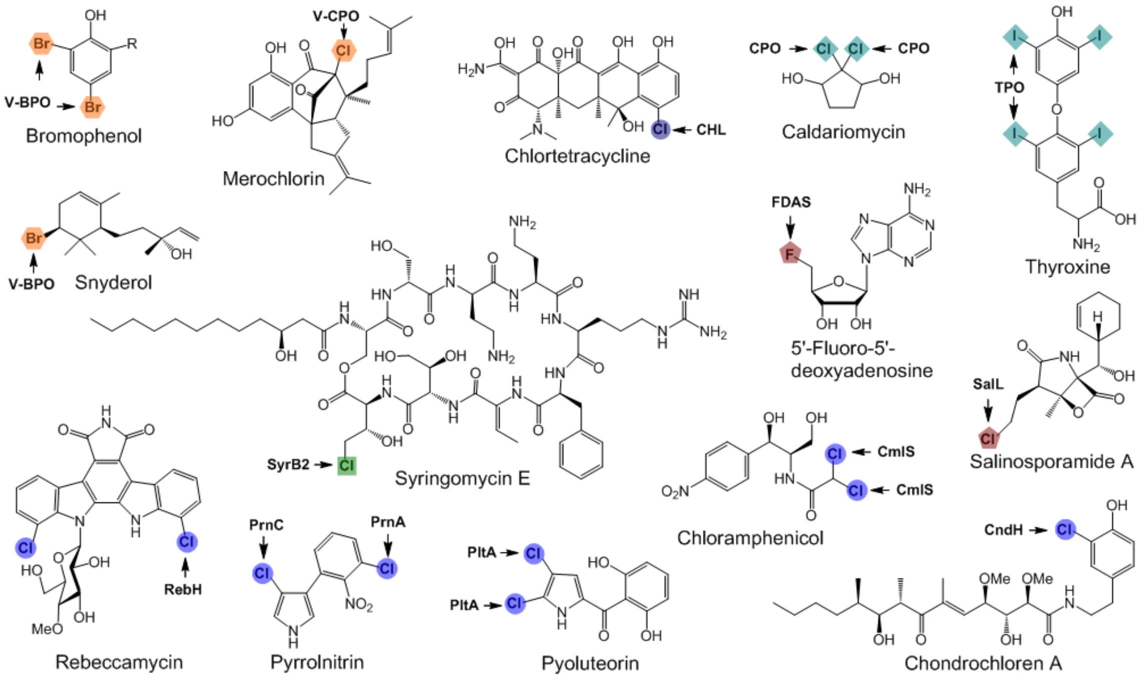

S1 Fig. Examples of halogenated natural products with their proposed specific halogenating enzymes. The colored markers indicate different halogenating enzyme families, and the shapes are in accord with that of the phylogenetic tree figures.
