# Supplementary material for: Independent Evolution of Six Families of Halogenating Enzymes
Source: PLoS One. 2016 May 6;11(5):e0154619. doi: 10.1371/journal.pone.0154619 (PMC4859513; doi:10.1371/journal.pone.0154619)
Supplement: S2 Fig — (PDF) [file pone.0154619.s002.pdf]

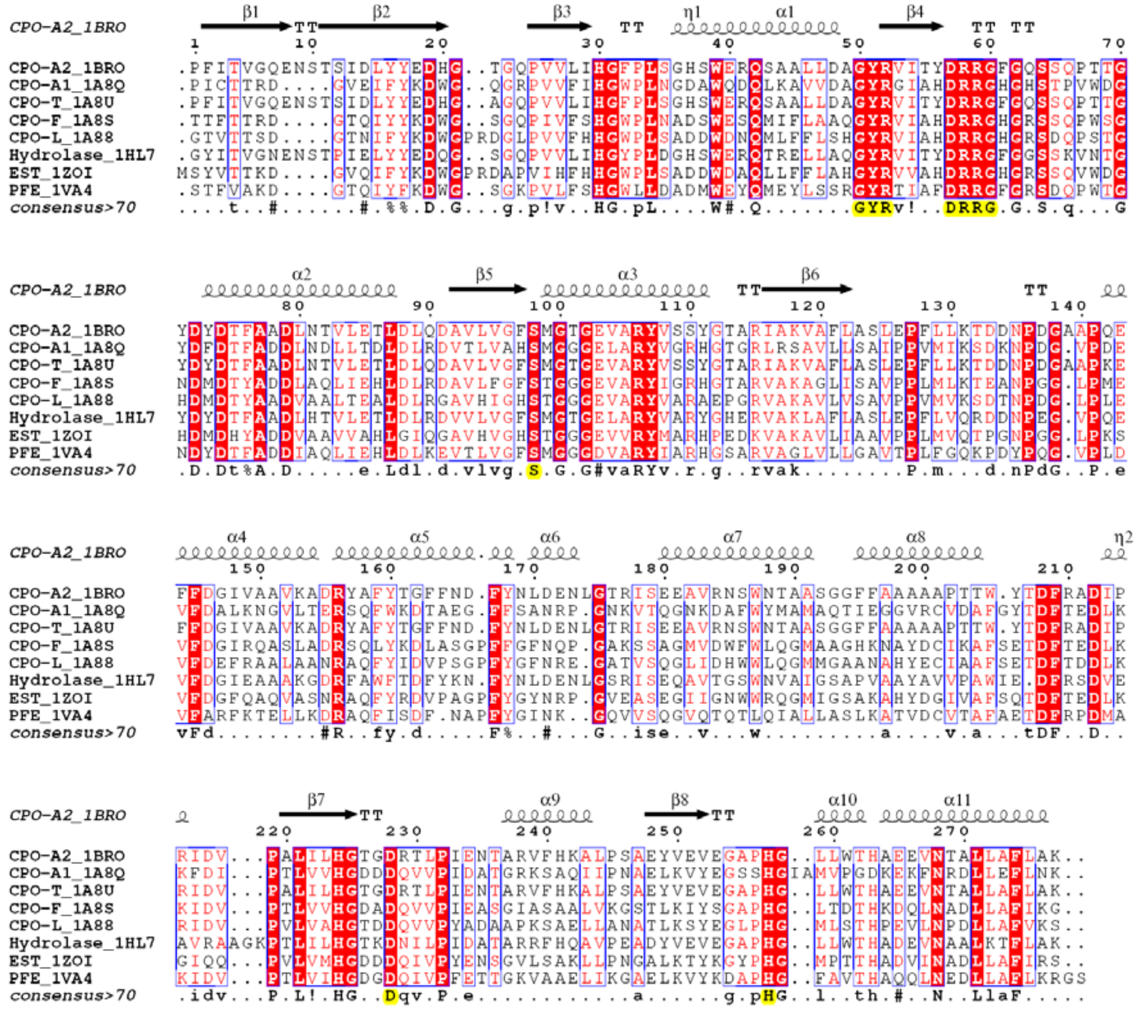

S2 Fig. Multiple sequence alignment of the cofactor-free HPO and the α/β hydrolases. The conserved residues include the GYR and DRRG motifs, the catalytic triad (S98-D228-H257), and the residues (HG, D-G-G-S and G-S-G-G) for structure arrangement and stabilization.
