# Supplementary figures and images for: Independent Evolution of Six Families of Halogenating Enzymes
Source: PLoS One. 2016 May 6;11(5):e0154619. doi: 10.1371/journal.pone.0154619 (PMC4859513; doi:10.1371/journal.pone.0154619)

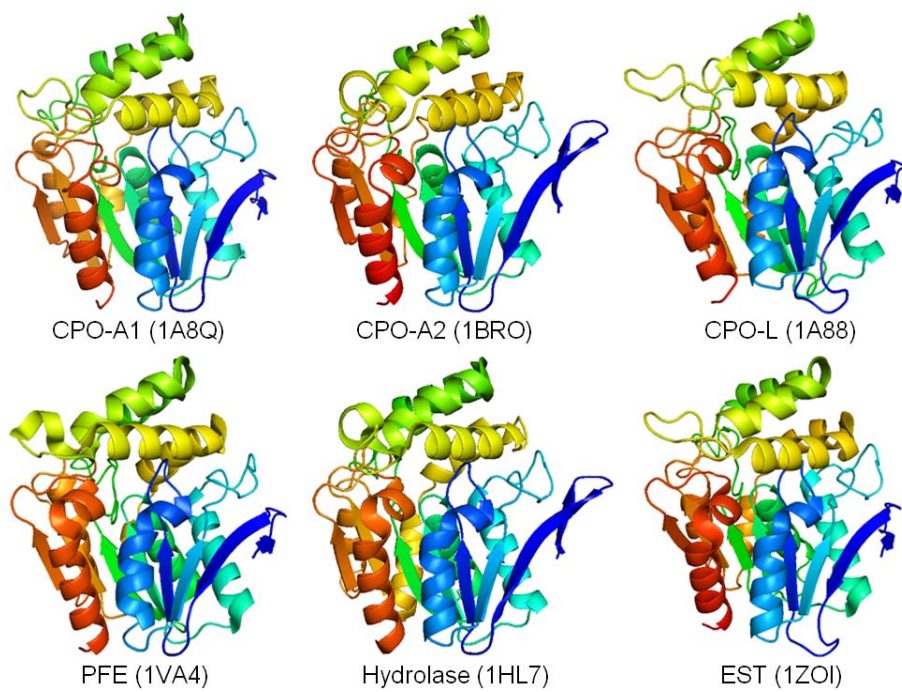

**S3 Fig. Structure comparison of the cofactor-free HPO with the  $\alpha/\beta$  hydrolases.**

Supplement: S3 Fig — (PDF) [file pone.0154619.s003.pdf]

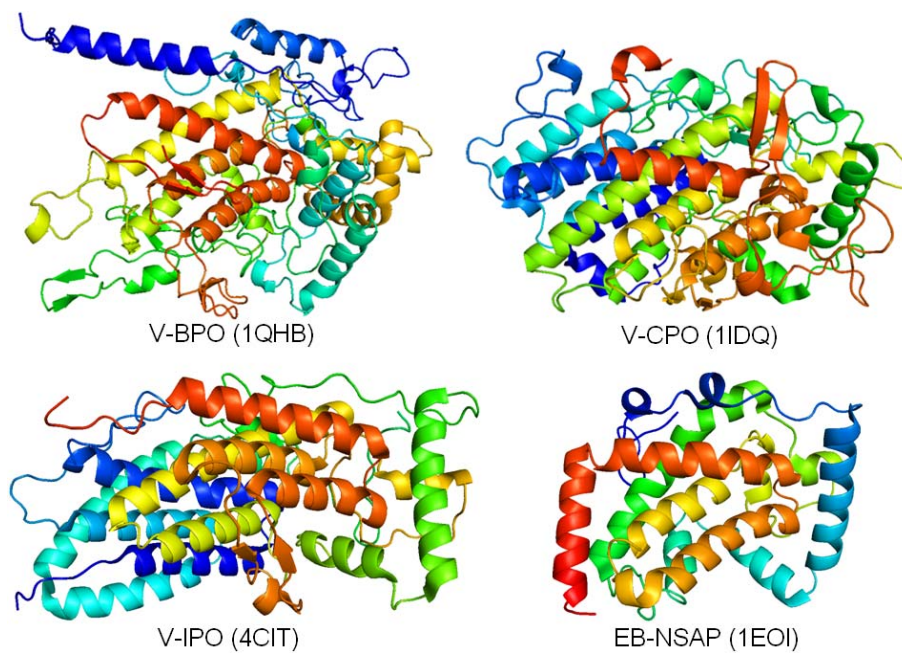

**S6 Fig. Structure comparison of the V-HPO and the acid phosphatase.**

Supplement: S6 Fig — (PDF) [file pone.0154619.s006.pdf]

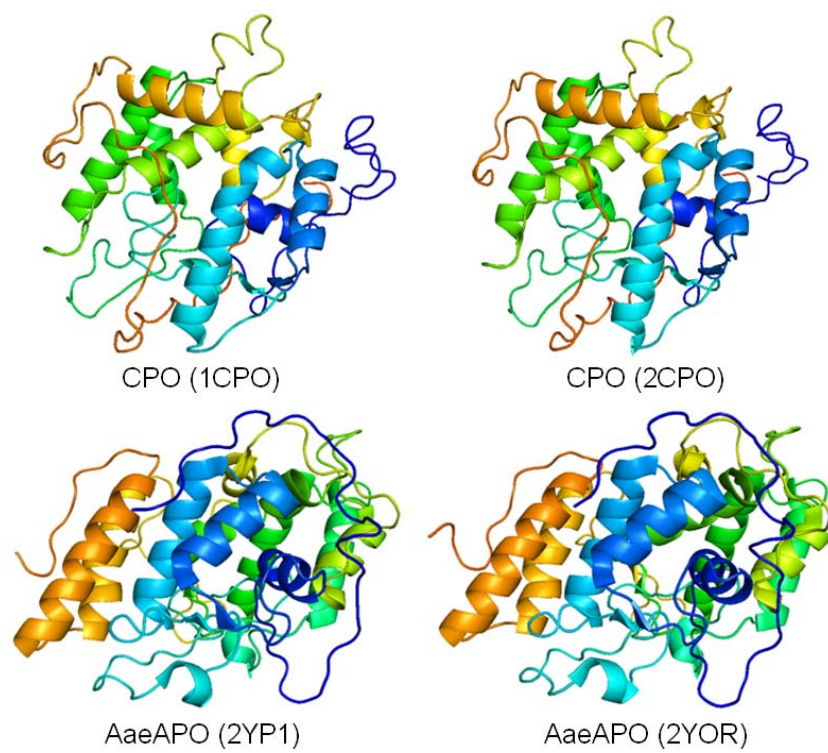

**S8 Fig. Structure comparison of the HI-HPO and the peroxidases.**

Supplement: S8 Fig — (PDF) [file pone.0154619.s008.pdf]

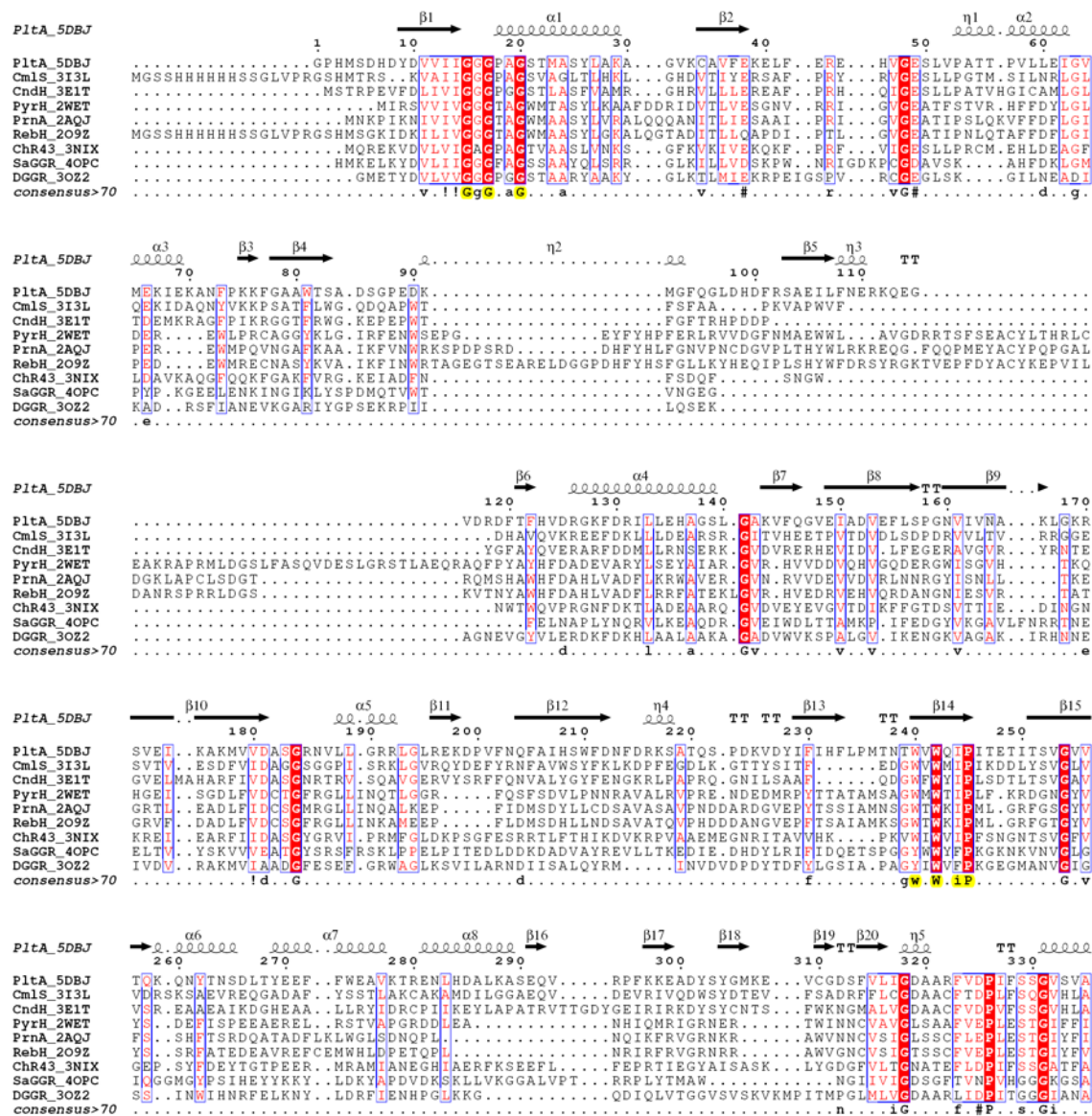

Supplement: S11 Fig — (PDF) [file pone.0154619.s011.pdf]

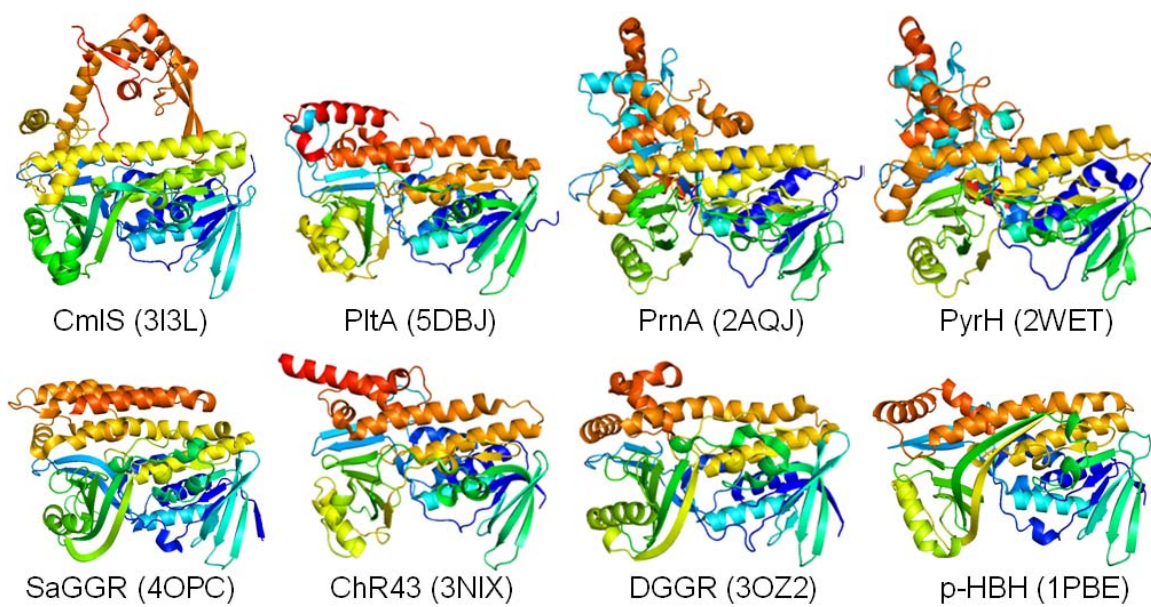

**S12 Fig. Structure comparison of the F-HG and the oxidoreductases.**

Supplement: S12 Fig — (PDF) [file pone.0154619.s012.pdf]

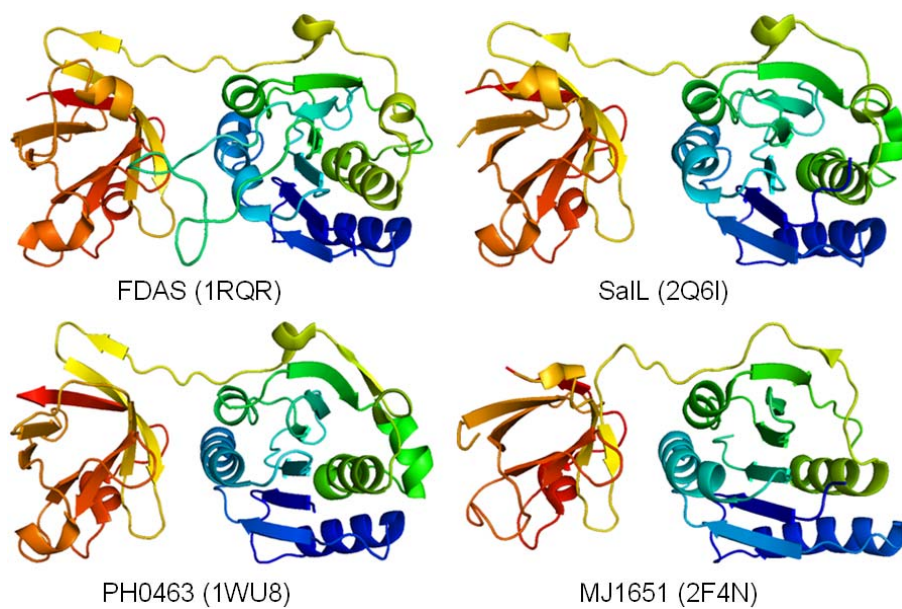

**S14 Fig. Structure comparison of the S-HG and the SAM hydroxide adenosyltransferases.**

Supplement: S14 Fig — (PDF) [file pone.0154619.s014.pdf]
