# Supplementary material for: Independent Evolution of Six Families of Halogenating Enzymes
Source: PLoS One. 2016 May 6;11(5):e0154619. doi: 10.1371/journal.pone.0154619 (PMC4859513; doi:10.1371/journal.pone.0154619)
Supplement: S4 Fig — (PDF) [file pone.0154619.s004.pdf]

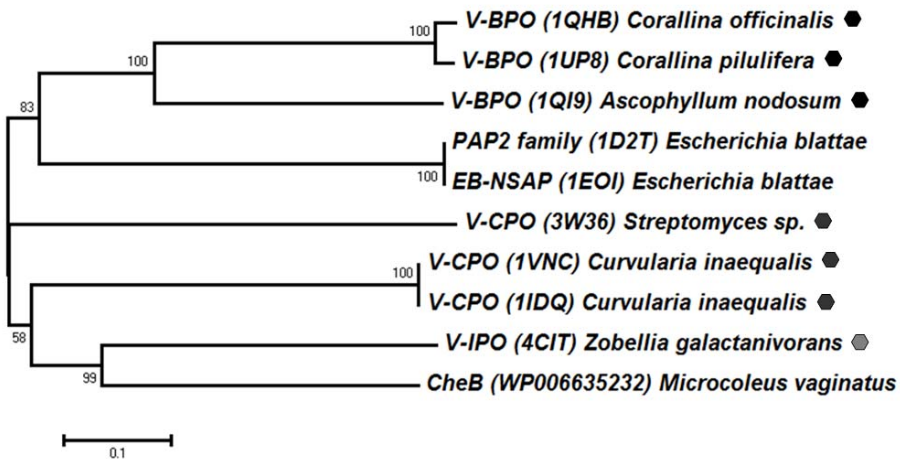

S4 Fig. Evolutionary relationships between the V-HPO and the acid phosphatases. The phylogenetic tree was reconstructed using the Neighbor-Joining method. The representative V-HPO enzymes are marked (⬣).
