# Supplementary material for: Independent Evolution of Six Families of Halogenating Enzymes
Source: PLoS One. 2016 May 6;11(5):e0154619. doi: 10.1371/journal.pone.0154619 (PMC4859513; doi:10.1371/journal.pone.0154619)
Supplement: S5 Fig — (PDF) [file pone.0154619.s005.pdf]

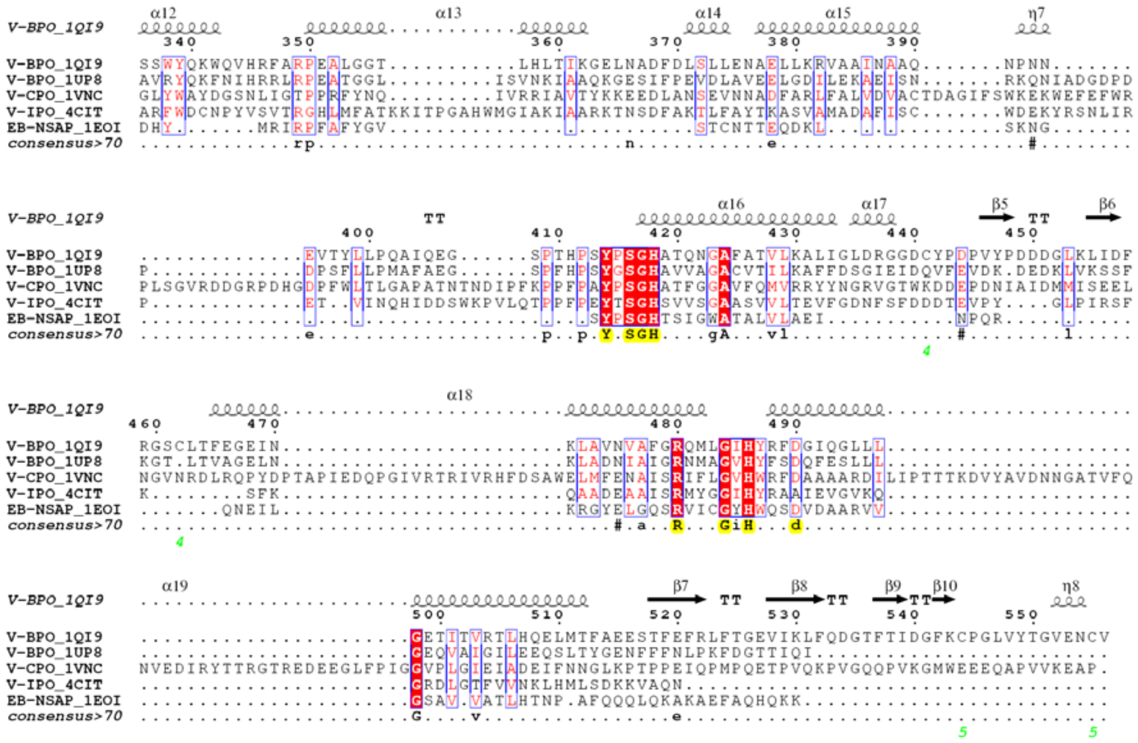

S5 Fig. Multiple sequence alignment of the V-HPO and the acid phosphatases. The conserved vanadate coordination sites RP, Y-SGH, and R-G-H-D are highlighted.
