# Supplementary material for: Independent Evolution of Six Families of Halogenating Enzymes
Source: PLoS One. 2016 May 6;11(5):e0154619. doi: 10.1371/journal.pone.0154619 (PMC4859513; doi:10.1371/journal.pone.0154619)
Supplement: S7 Fig — (PDF) [file pone.0154619.s007.pdf]

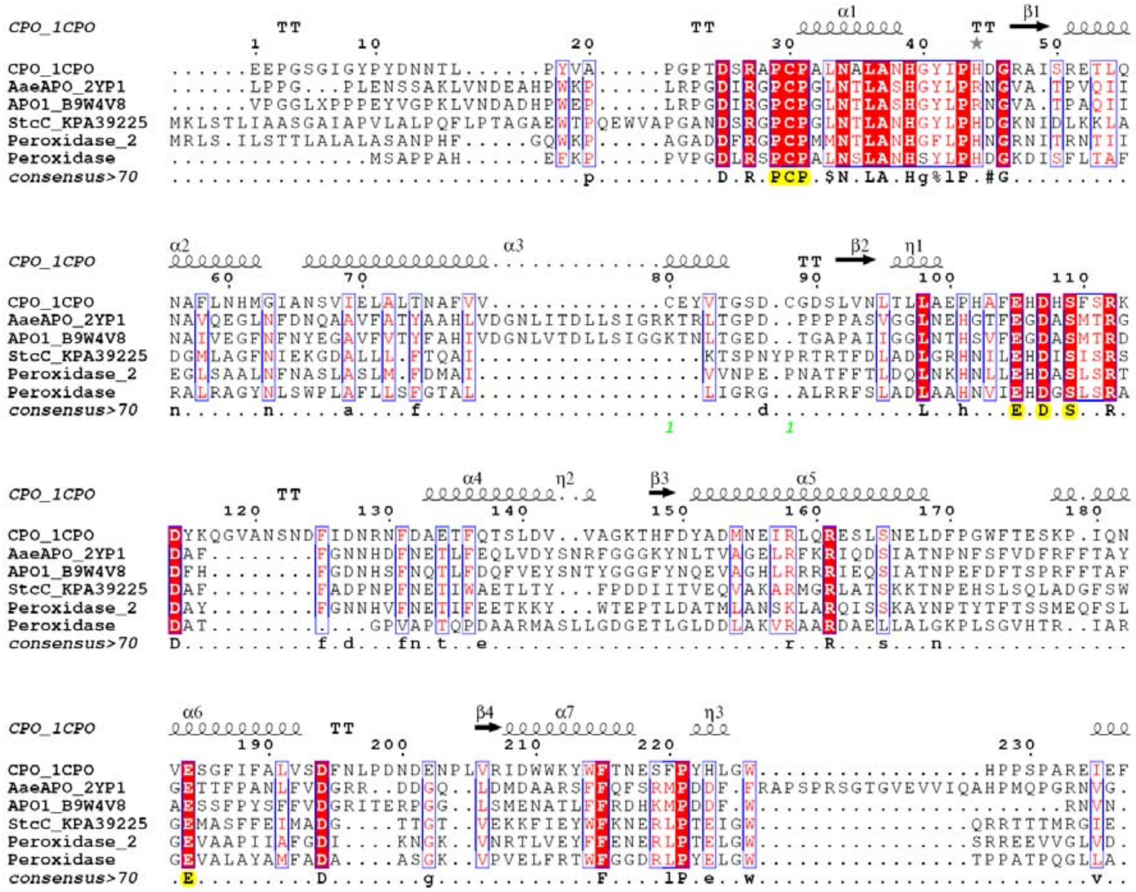

S7 Fig. Multiple sequence alignment of the HI-HPO and the peroxidases. The conserved proximal heme-binding motif (D-R-PCP-N-LA-H), the E-D-S motif for heme propionates, and the acid-base catalyst E residue in the distal heme pocket are highlighted.
