# Supplementary material for: Independent Evolution of Six Families of Halogenating Enzymes
Source: PLoS One. 2016 May 6;11(5):e0154619. doi: 10.1371/journal.pone.0154619 (PMC4859513; doi:10.1371/journal.pone.0154619)
Supplement: S9 Fig — (PDF) [file pone.0154619.s009.pdf]

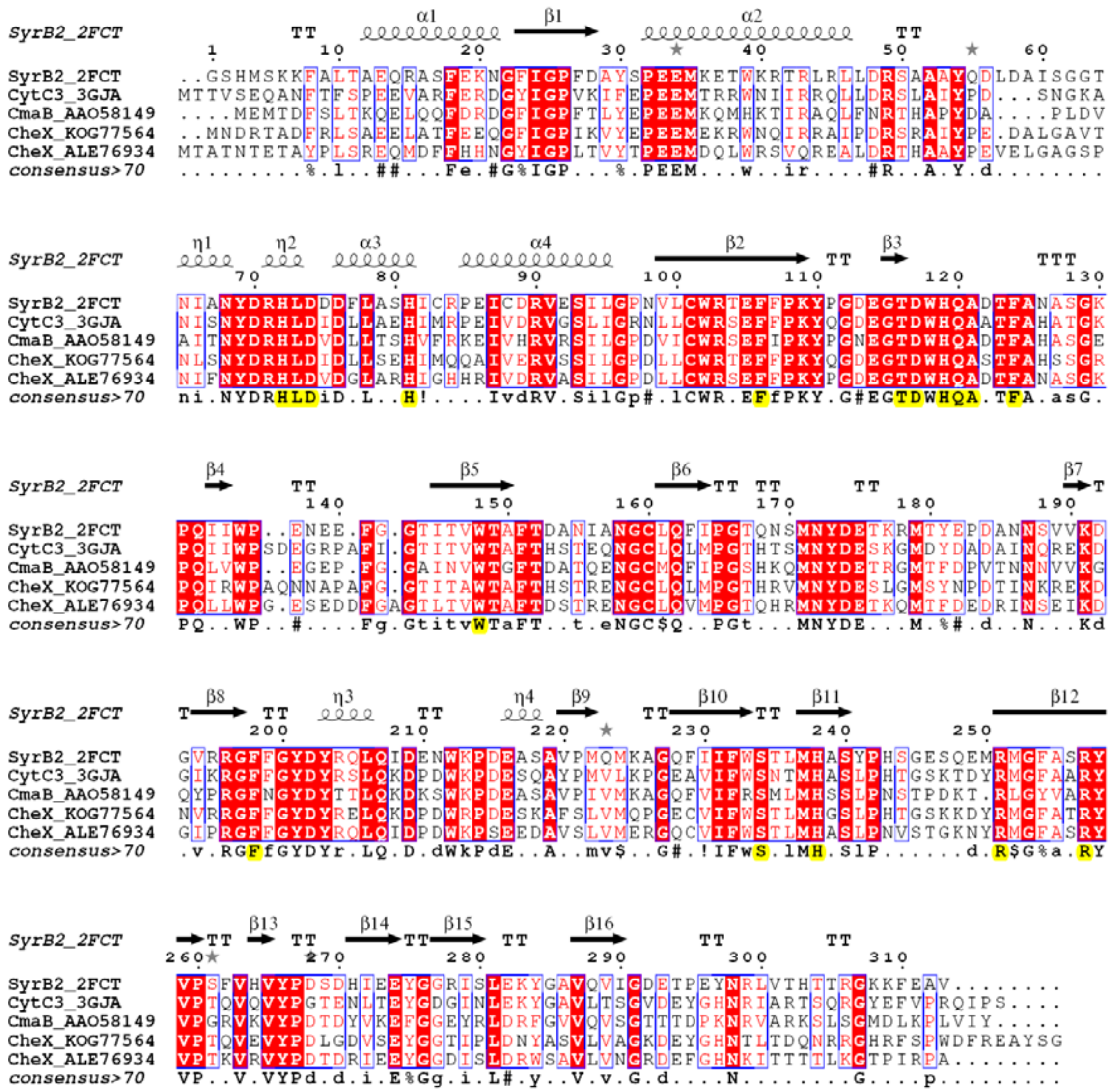

S9 Fig. Multiple sequence alignment of the NI-HG and the chemotaxis phosphatases. The conserved motifs (HLD-H and HQA-H) and the active site residues (H, F, R and S) in a hydrophobic pocket are highlighted.
