# Supplementary material for: Independent Evolution of Six Families of Halogenating Enzymes
Source: PLoS One. 2016 May 6;11(5):e0154619. doi: 10.1371/journal.pone.0154619 (PMC4859513; doi:10.1371/journal.pone.0154619)
Supplement: S10 Fig — (PDF) [file pone.0154619.s010.pdf]

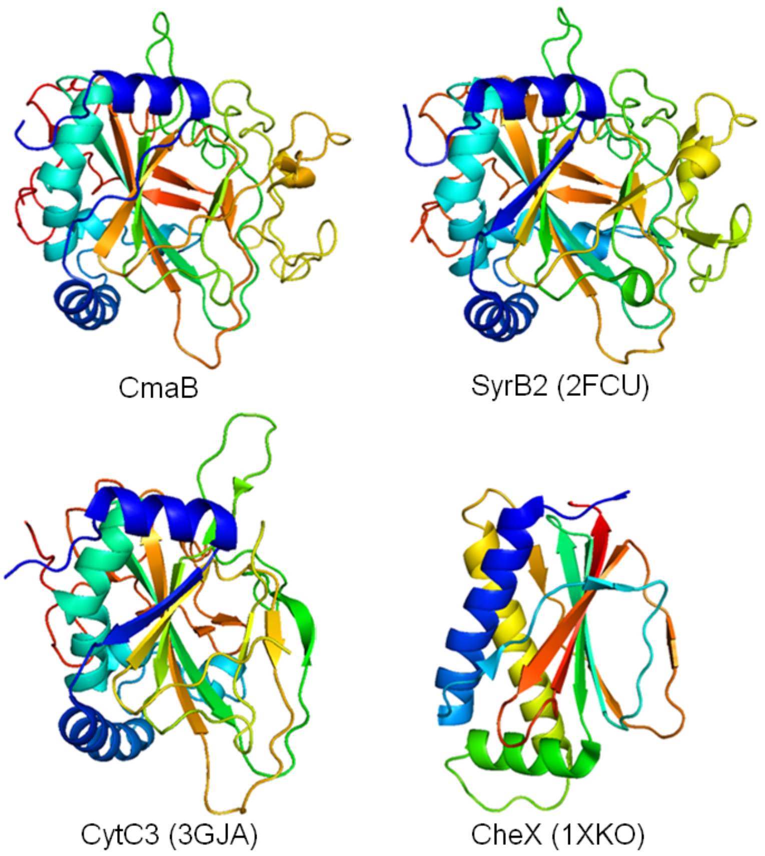


**S10 Fig. Structure comparison of the NI-HG and the chemotaxis phosphatase.** The structure of CmaB (AAO58149) from *Pseudomonas syringae* was predicted based on the crystal structure of SyrB2 (2FCU), using the SWISS-MODEL homology-modeling server.
