# Supplementary material for: Independent Evolution of Six Families of Halogenating Enzymes
Source: PLoS One. 2016 May 6;11(5):e0154619. doi: 10.1371/journal.pone.0154619 (PMC4859513; doi:10.1371/journal.pone.0154619)
Supplement: S13 Fig — (PDF) [file pone.0154619.s013.pdf]

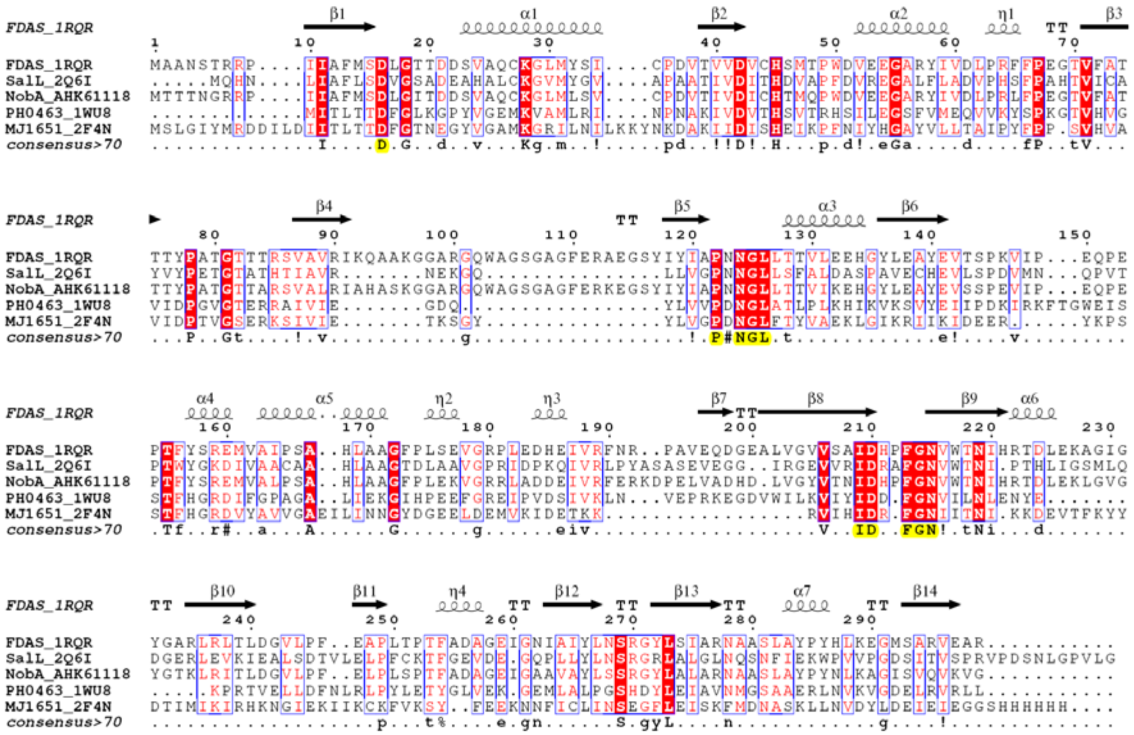

S13 Fig. Multiple sequence alignment of the S-HG and the SAM hydroxide adenosyltransferases. Two motifs (PxNGL and IDxxFGN) and several active site residues (D16 and N215) are conserved.
