# Supplementary material for: Independent Evolution of Six Families of Halogenating Enzymes
Source: PLoS One. 2016 May 6;11(5):e0154619. doi: 10.1371/journal.pone.0154619 (PMC4859513; doi:10.1371/journal.pone.0154619)
Supplement: S1 Table — (PDF) [file pone.0154619.s015.pdf]

S1 Table. Six families of halogenating enzymes.

**Halogenating enzyme families**	**Cofactors**	**Halogenating enzymes (PDB code)**	**Sources**
Cofactor-free haloperoxidases (HPO)	Free	CPO-A1 (1A8Q)	*Streptomyces aureofaciens*
		CPO-A2 (1BRO)	*Streptomyces aureofaciens*
		CPO-T (1A7U)	*Streptomyces aureofaciens*
		CPO-L (1A88)	*Streptomyces lividans*
		CPO-F (1A8S)	*Pseudomonas fluorescens*
Vanadium-dependent haloperoxidases (V-HPO)	Vanadate, H_2O_2	V-CPO (1VNC)	*Curvularia inaequalis*
		V-BPO (1QI9)	*Ascophyllum nodosum*
		V-BPO (1UP8)	*Corallina pilulifera*
		V-IPO (4CIT)	*Zobellia galactanivorans*
		V-CPO (3W36)	*Streptomyces* sp.
Heme iron-dependent haloperoxidases (HI-HPO)	Heme, H_2O_2	CPO (1CPO)	*Caldariomyces fumago*
		MPO (1CXP)	*Homo sapiens*
		LPO (2IKC)	*Ovis aries*
Non-heme iron-dependent halogenases (NI-HG)	Fe^{2+}, O_2 , α-KG	CmaB (AAO58149)	*Pseudomonas syringae*
		SyrB2 (2FCT)	*Pseudomonas syringae*
		CytC3 (3GJA)	*Streptomyces* sp.
		CurA-Hal (3NNJ)	*Lyngbya majuscula*
Flavin-dependent halogenases (F-HG)	$FADH_2$, O_2	PyrH (2WET)	*Streptomyces rugosporu*
		RebH (2O9Z)	*Lechevalieria aerocolonigenes*
		PrnA (2AQJ)	*Pseudomonas fluorescens*
		PltA (5DBJ)	*Pseudomonas fluorescens*
		CndH (3E1T)	*Chondromyces crocatus*
		CmlS (3I3L)	*Streptomyces venezuelae*
SAM-dependent halogenases (S-HG)	SAM	FDAS (1RQR)	*Streptomyces cattleya*
		SalL (2Q6K)	*Salinispora tropica*
